# Impact of Prolonged Cessation of Organized Team Training Due to the COVID-19 Pandemic on the Body Composition of Japanese Elite Female Wheelchair Basketball Athletes

**DOI:** 10.3390/jcm12093231

**Published:** 2023-04-30

**Authors:** Ryu Ishimoto, Hirotaka Mutsuzaki, Kaori Tachibana, Yukiyo Shimizu, Yasushi Hada

**Affiliations:** 1 Graduate School of Comprehensive Human Sciences, University of Tsukuba, Tsukuba 305-8575, Japan; 2 Department of Physical Medicine and Rehabilitation, Ibaraki Prefectural University of Health Sciences Hospital, Ami 300-0331, Japan; 3 Department of Orthopedic Surgery, Ibaraki Prefectural University of Health Sciences Hospital, Ami 300-0331, Japan; 4 Center for Medical Science, Ibaraki Prefectural University of Health Sciences, Ami 300-0394, Japan; 5 Department of Physical Therapy, School of Healthcare, Ibaraki Prefectural University of Health Sciences, Ami 300-0394, Japan; 6 Department of Rehabilitation Medicine, Institute of Medicine, University of Tsukuba, Tsukuba 305-8575, Japan

**Keywords:** female, wheelchair basketball, body composition, whole-body dual-energy X-ray absorptiometry, detraining, physical inactivity, COVID-19, lockdown

## Abstract

Studies on the effects of training confinement on athletes with physical impairments are limited. Hence, in this retrospective cohort study, we aimed to investigate the impact of prolonged cessation of organized team training due to the coronavirus disease 2019 pandemic on the body composition of elite female Japanese basketball athletes. Fourteen female wheelchair basketball athletes (aged ≥20 years) were enrolled. The primary outcomes were lean and adipose indices measured using whole-body dual-energy X-ray absorptiometry. The impact of prolonged organized team training cessation on body composition was investigated by comparing the body composition at baseline and post-training confinement. A reduced whole-body lean mass (*p* = 0.038) and percent lean mass (*p* = 0.022), as well as an increased percent body fat (*p* = 0.035), were observed after the confinement period. The regional analysis revealed reduced percent lean and increased percent fat masses in the trunk (*p* = 0.015 and *p* = 0.026, respectively) and upper limbs (*p* = 0.036 and *p* = 0.048, respectively). In conclusion, prolonged organized team training cessation reduced lean mass and increased body fat percentage, primarily in the trunk and upper limbs. Individualized training programs targeting these body regions should be implemented to improve body composition and physical conditions in athletes during and after prolonged cessation of organized team training.

## 1. Introduction

The coronavirus disease 2019 (COVID-19) pandemic deprived athletes worldwide of opportunities to participate in sports in an accustomed environment. In response to the spread of the infection, the Japanese government declared a state of emergency on 7 April 2020 in seven prefectures; the same was implemented nationwide on 16 April 2020 [1]. This state of emergency required that citizens stay home as much as possible. Consequently, many competitive events across various sports were canceled or postponed. Furthermore, although athletes were allowed to exercise outdoors, social distancing and the closure of gymnasiums, training facilities, and track-and-fields restricted athletes from practicing [2]. During this time, notable changes were reported in the training routines, training frequencies, motivation, training efforts, perceived distress, and dietary habits of the athletes [3,4,5,6,7,8,9]. The physical and psychological alterations experienced by the athletes during this period may have consequently led to a decline in their physical condition, potentially heightening the risk of future injuries upon returning to sports [10].

Recent studies reported the detrimental effects of the COVID-19 pandemic-induced confinement on the performance and body composition of athletes [2,11,12,13,14,15,16,17,18]. For example, Grazioli et al. [11] reported that 63 days of quarantine increased body mass and fat mass and decreased physical performance in professional Brazilian soccer players. Furthermore, Alvurdu et al. [12] reported that the absence of organized training for 15 weeks due to stay-at-home orders resulted in a detrimental effect on body fat percentage, neuromuscular performance, and aerobic capacity in Turkish youth soccer players, and Yasuda et al. [2] reported increased fat mass in elite female Japanese fencers after two months of the stay-at-home order.

Athletes with physical impairments were expected to be influenced by the COVID-19 pandemic; however, the impact may be greater than that on those without physical impairments [19,20,21]. Indeed, research investigating the effects of training confinement due to the COVID-19 pandemic on elite paralympic swimmers estimated a decrease in upper-body strength and power [22]. Conversely, a study investigating the effect of the COVID-19 pandemic on sedentary behavior, fitness, and diet of elite para-cyclists/para-triathletes reported increased sedentary screen time, but no differences in training volume, intensity, dietary intake, and fitness (as measured by power output) [23]. Furthermore, another study reported maintenance of sprint, agility, and dribbling time and improvement of countermovement jump height by the self-training programs during the COVID-19 mandatory lockdown in international football players with cerebral palsy [24].

Although the impact of the COVID-19 pandemic on athletes might vary depending on sports, disability, and the extent of public health interventions in their respective countries [25], there is no research to date that reported its impact on wheelchair basketball athletes. Furthermore, no study to our knowledge investigated the effect of the COVID-19 pandemic on the body composition of athletes with physical impairment. The lifestyle of athletes, including training routines, dietary habits, and psychological status, has changed during the COVID-19 pandemic, and this change might cause alternations in the body composition of athletes. The findings will likely help coaches, physical trainers, and team doctors to better care for the health of their athletes, to allow for their safe return to sports, and to devise optimal strategies to counteract the detrimental changes. Therefore, in this study, we aimed to investigate the effect of organized team training cessation due to the COVID-19 pandemic on the body composition of elite female wheelchair basketball players.

In response to the spread of COVID-19, the training camps and organized team training for the female Japanese wheelchair basketball athletes who were candidates for the international games in 2020 were canceled for six months (from February to August 2020) in accordance with the declaration of a state of emergency. During this time, athletes were requested to self-train at home or outdoors, with limited support from team members. Therefore, the body composition of athletes with physical impairment was hypothesized to undergo detrimental changes, resulting in reduced lean and increased adipose indices.

## 2. Materials and Methods

### 2.1. Participants

Elite female wheelchair basketball athletes (aged ≥20 years) who were potential candidates for the International Wheelchair Basketball Games in 2020 underwent medical checkups in April 2019 and September 2020. These medical checkups were conducted at the Ibaraki Prefectural University of Health Sciences Hospital, Japan. Athletes who underwent whole-body dual-energy X-ray absorptiometry (DXA) in both consecutive years were enrolled in this study. Those with missing data were excluded from the study.

**Figure 1:**
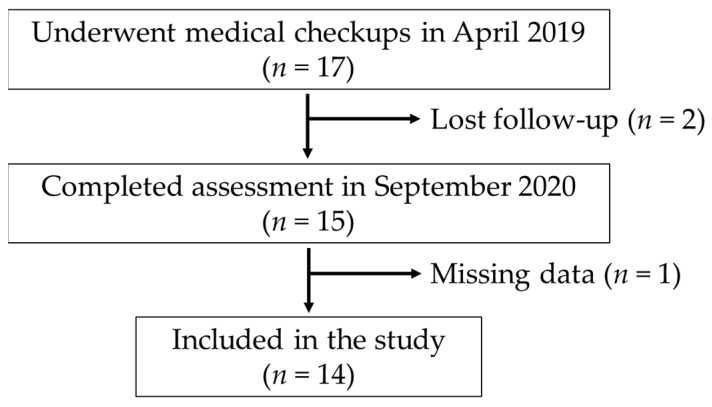
Flowchart of study participants.

Figure 1 shows the recruitment flowchart. Seventeen athletes underwent whole-body DXA in April 2019. Fifteen out of the seventeen participants underwent whole-body DXA in the consecutive year. Participants with missing basic information data (*n* = 1) were excluded from the analysis, and thus 14 participants were included in the study. The mean age of the participants was 29.2 ± 6.7 years. All participants had been playing wheelchair basketball at the national level for at least two years and did not have any acute or progressive health conditions. In addition, none of the participants were diagnosed with COVID-19 infection during the study period.

### 2.2. Study Design and Data Collection

This retrospective cohort study was performed to examine the change of body composition between the pre- and post-training confinement. Basic information, including age, underlying health conditions, disability, and International Wheelchair Basketball Federation (IWBF) classification [26], was obtained through medical chart abstraction. In addition, participants were categorized based on their wheelchair use status, underlying health conditions, and IWBF classification [27]. The participants relying on wheelchairs solely for basketball were classified as wheelchair non-users and those dependent on the daily use of wheelchairs were classified as wheelchair users. The underlying health conditions were classified as neurological or skeletal disorders. The neurological disorders (*n* = 7) included spinal cord injury, spina bifida, and polyneuropathy. Underlying skeletal disorders (*n* = 7) included below-knee amputation, articular diseases, and tumors. Based on the IWBF classification, the participants were divided into two categories: players with an IWBF classification within 1.0–2.5 were termed the low-point players (group A), and those with a classification within 3.0–4.5 were termed the high-point players (group B). Anthropometric evaluations and body composition analysis using DXA were performed in April 2019 (baseline, T0) and September 2020 (post-confinement, T1). All assessments of the participants were conducted on the same day.

### 2.3. Outcome Measurements

The primary outcomes included lean and adipose indices measured using whole-body DXA. These were measured using the Hologic Software Inc. Horizon A models (Marlborough, MA, USA). Hologic APEX software (version 5.6.04) was used to acquire and analyze whole-body scans. Quality control checks were performed before the scans, as indicated by the manufacturer. The same densitometer was used for all the scans.

Height was measured in a standing position for wheelchair non-users and a supine position for wheelchair users using a tape measure. In cases where deformities prevented accurate measurements, the arm span was measured instead of the height using a tape measure [28]. Body mass was measured using an electronic scale (Tanita digital scale BWB-627, Tanita Corp., Tokyo, Japan). Participants who could not stand on the scale were weighed on a wheelchair scale (Yamato digital scale DP-7101PW, Yamato Scale Co., Ltd, Hyogo, Japan). Body mass index (BMI) was calculated as the body mass divided by the square of the height (kg/m^2^). The percentages of lean and fat mass were determined for the trunk, upper limbs, and lower limbs to normalize the individual body size differences between the groups [29].

### 2.4. Statistical Analysis

Data were expressed as mean (standard deviation) for parametric data, median (interquartile range) for nonparametric data, and numerical values (%) for categorical data. The Shapiro–Wilk test was performed to assess normality. Levene’s test was performed to evaluate the equality of variance. Depending on the variables, the *t*-test, Welch’s *t*-test, and Mann–Whitney U test were performed to compare the age, anthropometric (i.e., height, body mass, and BMI), and DXA-measured body composition outcomes of the participants between groups. Fisher’s exact test was performed to compare numerical values for categorical data; the prevalence of underlying health conditions, wheelchair use status, and IWBF classification were compared between groups. In addition, a paired *t*-test and Wilcoxon signed-rank test were performed to compare the body composition at baseline (T0) and after training confinement (T1). Phi (φ = W) was calculated to determine the effect size in Fisher’s exact test, while Cohen’s d (d) and effect size r were calculated to determine the effect size in the independent and paired sample *t*-test. The effect size values were interpreted as small (φ = 0.1, d = 0.2 and r = 0.1), medium (φ = 0.3, d = 0.5 and r = 0.3), and large (φ = 0.5, d = 0.8 and r = 0.5) [30]. Statistical significance was set at a *p*-value < 0.05. All statistical analyses were performed using IBM SPSS Statistics, version 28.0 (IBM, Tokyo, Japan).

### 2.5. Ethics

The study was conducted in accordance with the Declaration of Helsinki and the Ethical Guidelines for Medical and Health Research Involving Human Subjects. Owing to the retrospective nature of the study, we maintained an opt-out policy so that the eligible participants could withdraw from the study at any time. This was mentioned on the webpage of our hospital. Furthermore, the need for written informed consent was waived. The Ethics Committee of the Ibaraki Prefectural University of Health Sciences approved this study (approval no: e382; date of approval: 10 February 2023).

## 3. Results

### 3.1. Participants

The background characteristics of all participants and study groups are presented in Table 1. Based on the status of wheelchair use, the groups had similar variables, except for the IWBF classification, height, and body mass. Low-point players were more prevalent in wheelchair users than in wheelchair non-users; high-point players were more common in wheelchair non-users than in wheelchair users (*p* = 0.002 and φ = 0.87). The wheelchair non-users were significantly taller and heavier than the wheelchair users (*p* = 0.008, d = 2.34; and *p* = 0.002, d = 2.16, respectively). BMI was not statistically different between the groups.

Based on the underlying health condition, low-point players were found to be more prevalent in athletes with neurological disorders than in those with skeletal disorders; high-point players were more common in athletes with skeletal disorders than in those with neurological disorders (*p* = 0.015 and φ = 0.71). Athletes with skeletal disorders had higher body mass than those with neurological disorders (*p* = 0.013 and d = 1.55). The height and BMI of the athletes were not significantly different between the groups.

### 3.2. Lean Tissue and Fat Tissue Mass

The DXA-measured body compositions at baseline are presented in Table 2. Wheelchair users had significantly lower whole-body lean mass (*p* < 0.001 and d = 3.97), lower percent lean mass (*p* = 0.001 and d = 2.27), and higher percent body fat (*p =* 0.001 and d = 2.22) than wheelchair non-users. The regional analysis revealed that wheelchair users had a significantly lower lean mass in the trunk (*p =* 0.001 and d = 3.07) and lower limbs (*p* < 0.001 and d = 4.02) than wheelchair non-users. Percent lean mass showed the same trends (*p* = 0.008 and d = 1.62 in the trunk; *p* < 0.001 and d = 3.49 in lower limbs). Furthermore, wheelchair users demonstrated a higher percent fat mass in the trunk (*p =* 0.012 and d = 1.50) and lower limbs (*p* < 0.001 and d = 3.68) than wheelchair non-users. In addition, the visceral adipose tissue was higher in wheelchair users than in wheelchair non-users (*p* = 0.021 and d = 1.43). There was no significant difference in the lean tissue mass in the upper limbs or the subcutaneous adipose tissue mass between the groups. Concerning the underlying health conditions, athletes with neurological disorders demonstrated lower whole-body lean mass than those with skeletal disorders (*p* = 0.019 and d = 1.45). Regional analysis revealed that lean mass in the trunk was considerably lower in athletes with neurological disorders than in those with skeletal disorders (*p* = 0.010 and d = 1.64). No significant difference was found in the adipose indices between the groups.

Table 3 presents the intergroup comparison of the change in the DXA-measured body composition outcomes after training confinement. No significant differences were observed between the groups; therefore, confinement-induced effects on body composition were examined in all the participants.

Table 4 presents the anthropometric and DXA-measured body composition comparisons between baseline and post-training confinement. A significant reduction in whole-body lean mass (*p* = 0.038 and d = 0.62) and percent lean mass (*p* = 0.022 and r = 0.61), as well as a gain in percent body fat (*p* = 0.035 and r = 0.56), were found after confinement. Regional analysis revealed a significant reduction in percent lean mass in the trunk (*p* = 0.015 and d = 0.75) and upper limbs (*p =* 0.036 and d = 0.63). In contrast, the percentage of body fat was significantly higher after confinement in the trunk (*p =* 0.026 and r = 0.60) and upper limbs (*p =* 0.048 and d = 0.58) than at the baseline. No significant difference was found in the absolute value of fat tissue mass in kg.

## 4. Discussion

Studies on the impact of the COVID-19 pandemic-induced training confinement on athletes have been conducted across various sports; however, there are few reports on the effects of training confinement on athletes with physical impairments. To the best of our knowledge, this is the first study to examine the impact of prolonged organized team training cessation due to the COVID-19 pandemic on the body composition of elite female wheelchair basketball players. These findings are paramount for maintaining the health, nutrition, physical condition, and physical performance of athletes, particularly for those with physical impairments. Our results revealed two main observations: (1) notable differences were found in body composition between the groups at the baseline; (2) after training confinement, reduced lean mass and increased percent body fat were found, particularly in the trunk and upper limbs of the athletes, irrespective of the status of wheelchair use and underlying health conditions.

### 4.1. Body Composition Differences between the Groups at Baseline

At the baseline, notable differences were observed in body composition between the groups. The results revealed that wheelchair users exhibited a lower whole-body lean mass and higher body fat percentage than wheelchair non-users. Moreover, the regional analysis revealed that wheelchair users demonstrated significantly lower lean mass and higher percent fat mass in the trunk and lower limbs than wheelchair non-users, with the upper limbs unaffected. These results are consistent with those of previous studies, where a significantly lower whole-body lean mass was discovered in daily wheelchair users as compared to wheelchair non-users [27,31]. Conversely, the lean tissue mass in the upper limbs and the percent body fat between the groups contradicted findings obtained in previous studies. Willems et al. [31] assessed the body composition of 14 elite male wheelchair athletes from wheelchair basketball and rugby and reported significantly lower lean tissue mass in the upper limbs of wheelchair users compared to wheelchair non-users. However, Shimizu et al. [27] compared the body composition of 13 female wheelchair basketball players and revealed no significant difference in the lean mass of the upper limbs between the groups. Furthermore, the percentage of body fat was not significantly different between the groups in the two studies.

In addition, our results indicated that athletes with neurological disorders demonstrated lower whole-body lean and trunk lean mass than those with skeletal disorders; however, no considerable difference was discovered in adipose indices between the groups. These results are consistent with those of studies where individuals with spinal cord injuries had significantly lower lean mass than those with skeletal disorders [27] and athletes without disabilities [32]. However, some studies reported a statistically higher percent body fat in athletes with spinal cord injuries than in those with lower limb amputations [33] and healthy athletes [32].

These discrepancies may be attributed to differences in the characteristics of athletes. For example, in the study by Willems et al. [31], all wheelchair users had complete tetraplegia; however, wheelchair users in this study were players with paraplegia, which spares the motor function of the upper limbs [34]. Likewise, differences in age, sex, underlying disorders, training level, and types of sports might have influenced the results [35]. As both studies were conducted with limited sample sizes, further studies are needed to determine the attributes of body composition in athletes with physical impairments.

### 4.2. Impact of Prolonged Cessation of Organized Team Training on Body Composition

Physical activities exhibited a positive effect on the body composition of athletes. For example, a study reported a gain in fat-free soft tissue mass and a reduction in fat mass during the preseason training phase in Australian football [36]. In addition, a cohort study reported an increase in lean mass in the upper limbs and a reduction in fat mass in the whole body, trunk, and lower limbs of elite male wheelchair rugby players during regular training sessions [37]. Furthermore, research comparing the body composition of wheelchair athletes to non-athletic individuals with physical impairments revealed that wheelchair athletes demonstrated lower fat mass and percentage fat mass in the whole body and trunk than non-athletic individuals with physical impairments [38]. Conversely, training confinement was expected to initiate the opposite response.

Alvurdu et al. [12] investigated the impact of a 15-week organized training absence in 68 Turkish Super League youth soccer players and reported a detrimental effect on percent body fat, neuromuscular performance, and aerobic capacity. In addition, Grazioli et al. [11] investigated the impact of 63 days of COVID-19 quarantine on 23 male Brazilian professional soccer players and reported increased body mass, fat mass, and decreased sprint and countermovement jump performance compared to traditional off-season values. Furthermore, Yasuda et al. [2] investigated the effect of the state of emergency on the body composition of 43 elite Japanese fencers and reported an increase in fat mass in females; however, no changes were demonstrated in the male athletes. Our results are consistent with these studies. Following the confinement period, a significant increase in body fat percentage was observed, particularly in the trunk and upper limbs. In addition, a study investigating the effect of training hours on fat mass in wheelchair athletes reported that the percent body fat of the trunk and upper limbs was higher in athletes who exercised <7 h/week than in those who exercised ≥7 h/week [39]. Based on the path analysis, training load influenced the percentage of fat mass on the trunk and upper limbs.

The musculature of the trunk and upper limbs plays a vital role in wheelchair basketball [40,41,42]. In games, athletes are constrained in wheelchairs and perform complex wheelchair maneuvers, such as propelling, stopping, and changing direction quickly and repeatedly; additionally, technical ball skills (such as dribbling, passing, and shooting) are required. Furthermore, athletes must withstand collisions when they contact the wheelchairs of defenders. These movements require the musculature of the trunk and upper limbs. In our study, whole-body lean mass was reduced after confinement, and a reduced percentage of lean mass on the trunk and upper limbs was observed. These results suggest that the cessation of team training at the gymnasium might have reduced the training load, especially on the trunk and upper limbs, causing detrimental changes in these body regions.

Furthermore, the change in body composition outcomes was not different between the groups after confinement. This result supports the phenomenon that the change in physical fitness after the COVID-19 mandatory lockdown was not different between sports classes (based on the severity of impairment) in football players with cerebral palsy [24]. These results may reflect the fact that, regardless of their disabilities, athletes face similar challenges. For example, a study based on an online survey of over twelve thousand athletes worldwide reported that during the COVID-19-induced lockdown, less than 40% of athletes could maintain sport-specific training; 80% of athletes trained alone; and the training frequency, intensity, and duration were markedly reduced [5]. In addition, a survey of Paralympic athletes from 15 sports disciplines reported that only 5.4% of athletes had access to sports facilities and their weekly training hours were reduced by nearly half [43]. Furthermore, another study that investigated the effects of the COVID-19 pandemic on behaviors and psychological status of Olympic and Paralympic level athletes demonstrated that 48.7% of the athletes reported perceived tiredness more than normal, 47.9% had difficulty sleeping, and 62.9% had developed changes in their eating habits [4]. Social distancing measures have influenced athletes across sports disciplines and athletes with physical impairments are not an exception. Athletes with physical impairment might face common challenges and many of these factors might negatively influence their lifestyle and body composition of athletes.

Moreover, the closure of gymnasiums and training facilities might have impacted indoor sports more than outdoor sports, as some of the equipment used in indoor sports were not usable outdoors [25]. For example, in wheelchair basketball, athletes are in a wheelchair specifically designed for basketball. Some features of the wheelchair include a wider wheelbase with a camber for ease of turning and lateral stability, a front bumper for impact absorption in collisions, and back wheels for tipping/flipping prevention. This equipment allows safe wheelchair maneuvers on a flat and smooth surface. Without access to the gymnasium, wheelchair maneuvers with ball handling could not be practiced, which might have caused a reduction in the training load and a change in body composition in the trunk and upper limbs. Nevertheless, continuous support from coaches through online meetings during the confinement period might have prevented the deteriorative changes in the lifestyle of athletes, including diet, fitness, and psychological status; retained the motivation; and minimized the deterioration of body composition.

### 4.3. Limitations

This study had some limitations. First, this was a retrospective cohort study of elite female Japanese wheelchair basketball athletes with a small sample size, limiting the generalizability of our results. Moreover, the study dealt with a highly specific population of athletes competing at national/international competitive levels, and it was difficult for some athletes to reach the site for medical checks on the same day. Second, this study did not assess individual differences in dietary habits, physical activity level, energy balance, or psychological status during the study period. During the confinement period, athletes received support through online meetings from the coaches and were requested to self-train at home or outdoors; however, differences in physical impairment type, motivations to remain fit, psychological distress, and dietary habits might have varied among athletes, which might have influenced the outcomes. Third, due to the retrospective nature of the study, the baseline data were assessed approximately ten months prior to the cessation of the organized team training. Hence, the changes in body composition observed in this study was the cumulative effect of the non-COVID-19 pandemic period and COVID-19 pandemic period. Although changes in body composition in the non-COVID-19 period is assumed to be minimal, confounders might have influenced the results. Fourth, the statistical analyses were repeated in this study, and thus a type I error should be considered when interpreting the results. The effect sizes are illustrated to help the interpretation of data. Fifth, it was impossible to ensure an after-fasting and standardized hydration status before the DXA scan due to the retrospective nature of the study. Sixth, two different body mass scales were used to assess the body mass of the participants; thus, possible measurement errors should be considered when interpreting the data.

Despite these limitations, this is the first study to our knowledge to report the impact of training confinement due to the COVID-19 pandemic on the body composition of wheelchair basketball athletes with physical impairment. The strength of this study was that body composition assessments were performed consecutively using DXA, which allowed us to evaluate the whole-body and regional body composition of the participants, thereby allowing the determination of body composition change that occurred during the confinement period.

### 4.4. Practical Applications

Detrimental change in body composition was observed during the COVID-19 pandemic-induced training confinement in female wheelchair basketball athletes. Medical team doctors, physical trainers, and coaches should expect these body composition changes in athletes when returning to the regular training routine. Medical checkups, including body composition assessment, are recommended to evaluate individual health condition. Individualized training programs, especially focusing on the trunk and upper limb regions, may aid athletes gain injury resilience and allow for a safe return to sport following a prolonged period of training confinement. In addition, physical trainers, and coaches should seek optimal strategies to maintain the physical condition of athletes in the event of future pandemics. If possible, programs should include whole-body exercises in a wheelchair, involving wheelchair maneuvers and ball handling, eliciting the desired training stress on both the trunk and upper limbs. Furthermore, routine online meetings with physical trainers, nutritionists, coaches, and team members should be scheduled to support athletes improve their nutritional and psychological health.

Moreover, similar body composition changes are also expected during the off-season, after abrupt cessation of training (i.e., after injuries), and when athletes retire from their careers. In such situations, regular exercise and appropriate diet habits are encouraged as adipose tissue accumulation, especially in the trunk region, is known to be linked with multiple health problems, such as cardiovascular disease and metabolic syndrome [44,45].

In Japan, a female wheelchair basketball team has been supported by a multi-disciplinary support team, including medical team doctors, physical therapists, physical trainers, nutritionists, and coaches [27]. Routine body composition monitoring has helped identify those in need of nutritional and therapeutic support. Further research is warranted to examine the detraining effects and the optimal strategies for athletes with a physical impairment to maintain their physical condition, especially in challenging situations that jeopardize fitness.

## 5. Conclusions

Prolonged cessation of organized team training significantly affected the body composition of elite female basketball athletes. Loss of lean mass and gain in percent body fat were observed, especially in the trunk and upper limbs. Similar body composition changes are also expected in the event of future pandemics, during the off-season, after an abrupt cessation of training (i.e., after injuries), and when athletes retire from sports. Training programs targeting the trunk and upper limbs should be implemented to improve the body composition and physical conditions of the athletes during and after an extended cessation of organized team training. Future studies are needed to further investigate the detraining effects on athletes with physical impairment and to devise optimal strategies to counteract the detrimental physical changes.

## Figures and Tables

**Table 1:** Characteristics of the participants (at the baseline, T0).

	Entire		WC Non-Users		WC Users					Neurological		Skeletal				
	*n* = 14 (100%)		*n* = 8 (57%)		*n* = 6 (43%)					*n* = 7 (50%)		*n* = 7 (50%)				
	Mean	±SD	Mean	±SD	Mean	±SD	*p*		d	Mean or Median	±SD or (IQR)	Mean or Median	±SD or (IQR)	*p*		d/r
Age (y)	29.2	±6.7	31.9	±6.2	25.7	±6.0	0.084	a	1.02	27.9	±6.7	30.6	±6.9	0.469	a	0.40
Anthropometrics																
Height (cm)	155.8	±12.9	164.2	±4.5	144.6	±11.9	0.008	b *	2.34	150.2	±15.4	161.4	±7.1	0.107	a	0.93
Body mass (kg)	51.5	±7.4	56.1	±3.7	45.3	±6.4	0.002	a *	2.16	46.9	±7.4	56.0	±3.9	0.013	a *	1.55
BMI (kg/m^2^)	21.2	±1.9	20.9	±1.7	21.7	±2.2	0.444	a	0.43	20.9	(18.8–23.9)	22.2	(20.4–22.4)	0.535	c	0.19
	*n*	%	*n*	%	*n*	%	*p*		φ	*n*	%	*n*	%	*p*		φ
Underlying health conditions																
Neurological	7	(50%)	2	(25%)	5	(83%)	0.051	e	0.58	-		-		-		
Skeletal	7	(50%)	6	(75%)	1	(17%)				-		-		-		
WC use status																
WC non-users	8	(57%)	-		-		-			2	(29%)	6	(86%)	0.051	e	0.58
WC users	6	(43%)	-		-		-			5	(71%)	1	(14%)			
IWBF classification																
Low-point players (Classification 1.0–2.5)	8	(57%)	1	(13%)	6	(100%)	0.002	e *	0.89	6	(86%)	1	(14%)	0.015	e *	0.71
High-point players (Classification 3.0–4.5)	7	(50%)	7	(88%)	0	(0%)				1	(14%)	6	(86%)			

Values are mean ±SD or median (IQR), *n* (%). Abbreviations: WC—wheelchair; IWBF—International Wheelchair Basketball Federation; BMI—body mass index; SD—standard deviation; IQR—interquartile range. a—Student’s *t*-test; b—Welch’s test; c—Mann–Whitney U test; d—Cohen’s d; e—Fisher’s exact test; r—effect size r; φ—Phi. * *p* < 0.05.

**Table 2:** Body composition comparison between the groups based on wheelchair use status and underlying health condition at baseline (T0).

	WC Non-Users *n* = 8		WC Users *n* = 6					Neurological *n* = 7		Skeletal *n* = 7				
	Mean	±SD	Mean	±SD	*p*		d	Mean or Median	±SD or (IQR)	Mean or Median	±SD or (IQR)	*p*		d/r
Whole-body														
Lean mass (kg)	40.0	±2.1	28.0	±4.0	<0.001	a *	3.97	30.8	±6.8	38.9	±3.9	0.019	a *	1.45
% Lean mass	70.3	±5.0	61.4	±1.0	0.001	b *	2.27	62.2	(60.8–71.3)	66.2	(63.4–75.4)	0.128	c	0.43
Fat mass (kg)	14.8	±4.0	15.8	±2.5	0.590	a	0.20	14.5	±2.9	15.9	±3.9	0.441	a	0.43
% Fat mass	25.6	±5.2	34.6	±1.3	0.001	b *	2.22	34.6	(24.6–34.9)	29.5	(20.1–32.6)	0.259	c	0.33
Trunk														
Lean mass (kg)	20.7	±1.3	15.0	±2.4	0.001	b *	3.07	16.1	±3.3	20.4	±1.8	0.010	a *	1.64
% Lean mass	76.4	±5.5	69.5	±1.6	0.008	b *	1.62	72.6	±6.0	74.3	±5.2	0.598	a	0.29
Fat mass (kg)	5.9	±2.1	6.0	±1.1	0.878	a	0.09	5.4	±1.3	6.5	±1.9	0.220	a	0.69
% Fat mass	21.2	±5.6	27.8	±1.6	0.012	b *	1.50	24.6	±5.7	23.5	±5.5	0.702	a	0.21
VAT (cm^2^)	46.2	±13.0	65.0	±13.3	0.021	a *	1.43	57.7	±17.0	50.8	±15.1	0.443	a	0.42
SAT (cm^2^)	194.6	±61.5	240.7	±55.2	0.173	a	0.78	218.5	±68.8	210.2	±58.2	0.812	a	0.13
Upper limbs														
Lean mass (kg)	5.0	±0.3	4.9	±0.7	0.814	b	0.15	4.9	±0.6	5.0	±0.4	0.568	a	0.31
% Lean mass	69.1	±7.5	63.9	±2.9	0.106	b	0.86	66.6	±5.8	67.2	±7.3	0.871	a	0.09
Fat mass (kg)	2.0	±0.8	2.5	±0.5	0.201	a	0.73	2.2	±0.6	2.2	±0.8	0.950	a	0.03
% Fat mass	26.5	±7.7	31.9	±3.1	0.106	b	0.86	29.1	±6.0	28.5	±7.5	0.878	a	0.08
Lower limbs														
Lean mass (kg)	11.5	±1.1	5.3	±2.0	<0.001	a *	4.02	7.1	±3.6	10.6	±2.6	0.059	a	1.12
% Lean mass	63.5	±5.8	43.7	±5.6	<0.001	a *	3.49	50.2	±11.4	59.9	±10.2	0.119	a	0.90
Fat mass (kg)	5.8	±1.3	6.3	±1.1	0.521	a	0.36	5.9	±1.1	6.1	±1.3	0.714	a	0.20
% Fat mass	32.1	±5.8	53.6	±5.9	<0.001	a *	3.68	46.6	±12.2	36.0	±10.9	0.115	a	0.91

Values are mean ±SD or median (IQR). Abbreviations—WC, wheelchair; VAT—visceral adipose tissue; SAT—subcutaneous adipose tissue; SD—standard deviation; IQR—interquartile range. a—Student *t*-test; b—Welch test; c—Mann–Whitney U test; d—Cohen’s d; r—effect size r. * *p* < 0.05.

**Table 3:** Intergroup comparisons for the change (Δ) of the DXA-measured body composition outcome after training confinement.

	WC Non-Users		WC Users					Neurological		Skeletal				
	Δ		Δ					Δ		Δ				
	Mean or Median	±SD or (IQR)	Mean or Median	±SD or (IQR)	*p*		d/r	Mean or Median	±SD or (IQR)	Mean or Median	±SD or (IQR)	*p*		d/r
Body mass (kg)	−0.1	±2.0	0.4	±4.0	0.778	a	0.16	0.3	±3.7	−0.1	±2.2	0.783	a	0.15
BMI (kg/m^2^)	−0.1	±0.8	1.4	±3.6	0.341	b	0.65	1.1	±3.4	−0.1	±0.9	0.378	a	0.50
Body composition														
Whole-body														
Lean mass (kg)	−0.8	±1.0	−0.7	±1.6	0.796	a	0.14	−0.5	±1.4	−1.1	±1.0	0.386	a	0.48
% Lean mass	−1.3	±1.7	−2.0	±2.7	0.569	a	0.32	−1.7	±2.5	−1.5	±1.9	0.849	a	0.10
Fat mass (kg)	0.8	±1.5	1.0	±2.9	0.891	a	0.08	0.9	±2.7	0.9	±1.6	0.949	a	0.04
% Fat mass	1.3	±1.8	1.9	±3.0	0.632	a	0.27	1.6	±2.7	1.4	±2.0	0.864	a	0.09
Trunk														
Lean mass (kg)	−0.6	±0.5	−0.3	±1.1	0.539	a	0.34	−0.1	±0.9	−0.8	±0.6	0.127	a	0.88
% Lean mass	−1.8	±2.2	−2.9	±4.0	0.522	a	0.36	−2.7	±3.7	−1.8	±2.3	0.634	a	0.26
Fat mass (kg)	0.5	±0.8	0.	±1.8	0.747	a	0.18	0.8	±1.6	0.5	±0.9	0.700	a	0.21
% Fat mass	1.8	±2.2	2.8	±4.3	0.562	a	0.32	2.6	±4.0	1.9	±2.4	0.675	a	0.23
VAT (cm^2^)	2.8	±9.0	9.0	±23.2	0.559	b	0.37	7.6	±21.7	3.3	±9.2	0.638	a	0.26
SAT (cm^2^)	7.5	±24.9	10.1	±86.7	0.947	b	0.04	7.7	±78.5	9.5	±28.8	0.957	a	0.03
Upper limbs														
Lean mass (kg)	−0.2	(−0.3–0.04)	−0.03	(−0.4–[−0.2])	0.852	c	0.07	−0.2	±0.2	−0.0	±0.2	0.375	a	0.49
% Lean mass	−1.8	±1.2	−1.5	±4.0	0.856	a	0.10	−2.5	(−3.0–[−0.4])	−1.0	(−2.5–[−0.4])	0.535	c	0.19
Fat mass (kg)	0.08	(0.02–0.2)	0.1	(−0.1–0.4)	0.852	c	0.07	0.01	(0.04–0.1)	0.01	(0.02–0.3)	0.902	c	0.05
% Fat mass	1.7	±1.2	1.4	±4.1	0.866	a	0.09	2.4	(0.2–2.9)	0.9	(0.4–2.8)	0.620	c	0.15
Lower limbs														
Lean mass (kg)	−0.1	±0.6	−0.4	±0.5	0.415	a	0.46	−0.2	±0.5	−0.2	±0.6	0.980	a	0.01
% Lean mass	−0.6	±2.5	−1.8	±1.3	0.339	a	0.54	−1.1	±0.9	−1.2	±2.9	0.912	b	0.06
Fat mass (kg)	0.2	±0.6	0.1	±0.7	0.781	a	0.15	0.01	±0.6	0.2	±0.6	0.560	a	0.32
% Fat mass	0.6	±2.5	1.7	±1.3	0.373	a	0.50	1.0	±1.0	1.2	±2.9	0.887	b	0.08

Values are mean ±SD or median (IQR). Abbreviations: WC—wheelchair; BMI—body mass index; VAT—visceral adipose tissue; SAT—subcutaneous adipose tissue; SD—standard deviation; IQR—interquartile range. a—Student *t*-test; b—Welch test; c—Mann–Whitney U test; d—Cohen’s d; r—effect size r.

**Table 4:** Body composition comparison between the baseline (T0) and post-training confinement (T1).

	T0		T1		Δ				
	*n* = 14		*n* = 14						
	Mean or Median	±SD or (IQR)	Mean or Median	±SD or (IQR)	Mean or Median	±SD or (IQR)	*p*		d/r
Body mass (kg)	51.5	±7.4	51.6	±7.6	0.1	±2.9	0.886	a	0.04
BMI (kg/m^2^)	21.2	±1.9	21.7	±2.5	0.5	±2.4	0.430	a	0.22
Body composition									
Whole-body									
Lean mass (kg)	34.8	±6.8	34.1	±6.7	−0.8	±1.2	0.038	a *	0.62
% Lean mass	64.0	(61.2–72.3)	62.3	(59.6–73.0)	−1.7	(−3.1–[−0.1])	0.022	b *	0.61
Fat mass (kg)	15.2	±3.4	16.1	±4.3	0.9	±2.1	0.135	a	0.43
% Fat mass	31.9	(23.6–34.9)	33.5	(22.9–35.9)	1.7	([−0.02]−2.9)	0.035	b *	0.56
Trunk									
Lean mass (kg)	18.2	±3.4	17.8	±3.4	−0.5	±0.8	0.059	a	0.55
% Lean mass	73.4	±5.5	71.2	±6.7	−2.3	±3.0	0.015	a *	0.75
Fat mass (kg)	5.9	±1.7	6.6	±2.3	0.6	±1.3	0.083	a	0.50
% Fat mass	26.0	(18.5–28.5)	27.4	(20.0–32.3)	2.4	(0.06–4.2)	0.026	b *	0.60
VAT (cm^2^)	54.2	±15.9	59.7	±23.2	5.4	±16.2	0.230	a	0.34
SAT (cm^2^)	214.4	±61.4	223.0	±87.1	8.6	±56.8	0.580	a	0.15
Upper limbs									
Lean mass (kg)	5.0	±0.5	4.9	±0.7	−0.1	±0.2	0.120	a	0.44
% Lean mass	66.9	±6.4	65.2	±6.6	−1.6	±2.6	0.036	a *	0.63
Fat mass (kg)	2.2	±0.7	2.3	±0.8	0.1	±0.3	0.147	a	0.41
% Fat mass	28.8	±6.6	30.4	±6.9	1.6	±2.7	0.048	a *	0.58
Lower limbs									
Lean mass (kg)	8.8	±0.3	8.6	±3.5	−0.2	±0.5	0.137	a	0.42
% Lean mass	55.1	±11.5	53.9	±12.6	−1.1	±2.1	0.063	a	0.54
Fat mass (kg)	6.0	±1.2	6.1	±1.4	0.1	±0.6	0.498	a	0.19
% Fat mass	41.3	±12.4	42.4	±13.4	1.1	±2.1	0.077	a	0.51

Values are mean ±SD or median (IQR). Abbreviations: BMI—body mass index; VAT—visceral adipose tissue; SAT—subcutaneous adipose tissue; SD—standard deviation; IQR—interquartile range. Δ represents the change in values between the baseline (T0) and post-training confinement (T1). A—paired *t*-test; b—Wilcoxon signed-rank test; d—Cohen’s d; r—effect size r. * *p* < 0.05.

## Data Availability

The data are not publicly available due to privacy concerns.
